# Aging-associated sensory decline and Alzheimer’s disease

**DOI:** 10.1186/s13024-024-00776-y

**Published:** 2024-12-04

**Authors:** Suji Hong, Seung-Hyun Baek, Mitchell K. P. Lai, Thiruma V. Arumugam, Dong-Gyu Jo

**Affiliations:** 1 The School of Pharmacy, Sungkyunkwan University, Suwon, 16419 Republic of Korea; 2 Department of Pharmacology, Yong Loo Lin School of Medicine, Singapore, 117600 Singapore; 3 Centre for Cardiovascular Biology and Disease Research, La Trobe Institute for Molecular Science, Department of Microbiology, Anatomy, Physiology and Pharmacology, School of Agriculture, Biomedicine and Environment, La Trobe University, Melbourne, 3086 Australia; 4 Biomedical Institute for Convergence at SKKU (BICS), Suwon, 16419 Republic of Korea; 5 Department of Health Sciences and Technology, SAIHST, Sungkyunkwan University, Seoul, 06355 Republic of Korea; 6 Institute of Quantum Biophysics, Sungkyunkwan University, Suwon, 16419 Republic of Korea

**Keywords:** Alzheimer's Disease (AD), Aging, Sensory impairments, Early biomarkers

## Abstract

Multisensory decline is common as people age, and aging is the primary risk of Alzheimer’s Disease (AD). Recent studies have begun to shed light on the possibility that age-related sensory decline could accelerate AD pathogenesis, or be a prodromal indicator of AD. Sensory impairments, specifically in taste and smell, often emerge before cognitive symptoms in AD, indicating their potential as early biomarkers. Olfactory dysfunction has been frequently associated with AD and may offer valuable insights into early detection. Hearing impairment is significantly associated with AD, but its causal impact on AD progression remains unclear. The review also discusses visual and tactile deficits in AD, including retinal thinning and changes in tactile perception, highlighting their links to disease progression. Focusing on molecular mechanisms, the review explores the roles of amyloid-β (Aβ) accumulation and tau protein pathology in sensory decline and their bidirectional relationship with AD. In summary, the evidence presented conclusively supports advocating for an integrated approach to understanding AD and sensory decline, to enhance early detection, implementing preventive strategies, and developing therapeutic interventions for AD. This approach underscores the significance of sensory health in addressing neurodegenerative diseases, particularly AD.

## Background

The interrelationship between aging, sensory deterioration, and onset of Alzheimer’s Disease (AD) forms a complex nexus within gerontology and neurodegenerative pathology research. Aging is marked by a progressive decline in physiological functions, affecting health, mobility, and quality of life. This decline, resulting from genetic, environmental, and lifestyle factors, leads to changes in various body systems, including the brain, cardiovascular, and musculoskeletal systems. One of the evident impacts of aging is on sensory function. Sensory decline, encompassing the deterioration of vision, hearing, taste, smell, and touch, affects the ability of older adults to interact with their environment. This decline is not just a loss of sensory acuity but also influences daily living, communication, and the ability to maintain social connections.

Age-related changes also lead to alterations in cognitive functions, such as processing speed, attention, memory, language skills, visuospatial abilities, and executive functioning [1]. In the context of the aging landscape, AD emerges as a major health challenge. Defined as a progressive neurodegenerative disorder, AD primarily manifests through cognitive decline and dementia, establishing it as the most prevalent form of dementia among the elderly. The pathogenesis of AD is complex and not entirely understood, involving genetic, environmental, and lifestyle factors. Central to AD are amyloid-β (Aβ) plaques and tau protein tangles in the brain, which interfere with cell communication and trigger inflammatory responses, leading to brain cell death [2, 3]. Notably, AD is marked by a loss of synaptic function and neuronal death, especially in regions critical for memory and cognition [4].

**Fig. 1:**
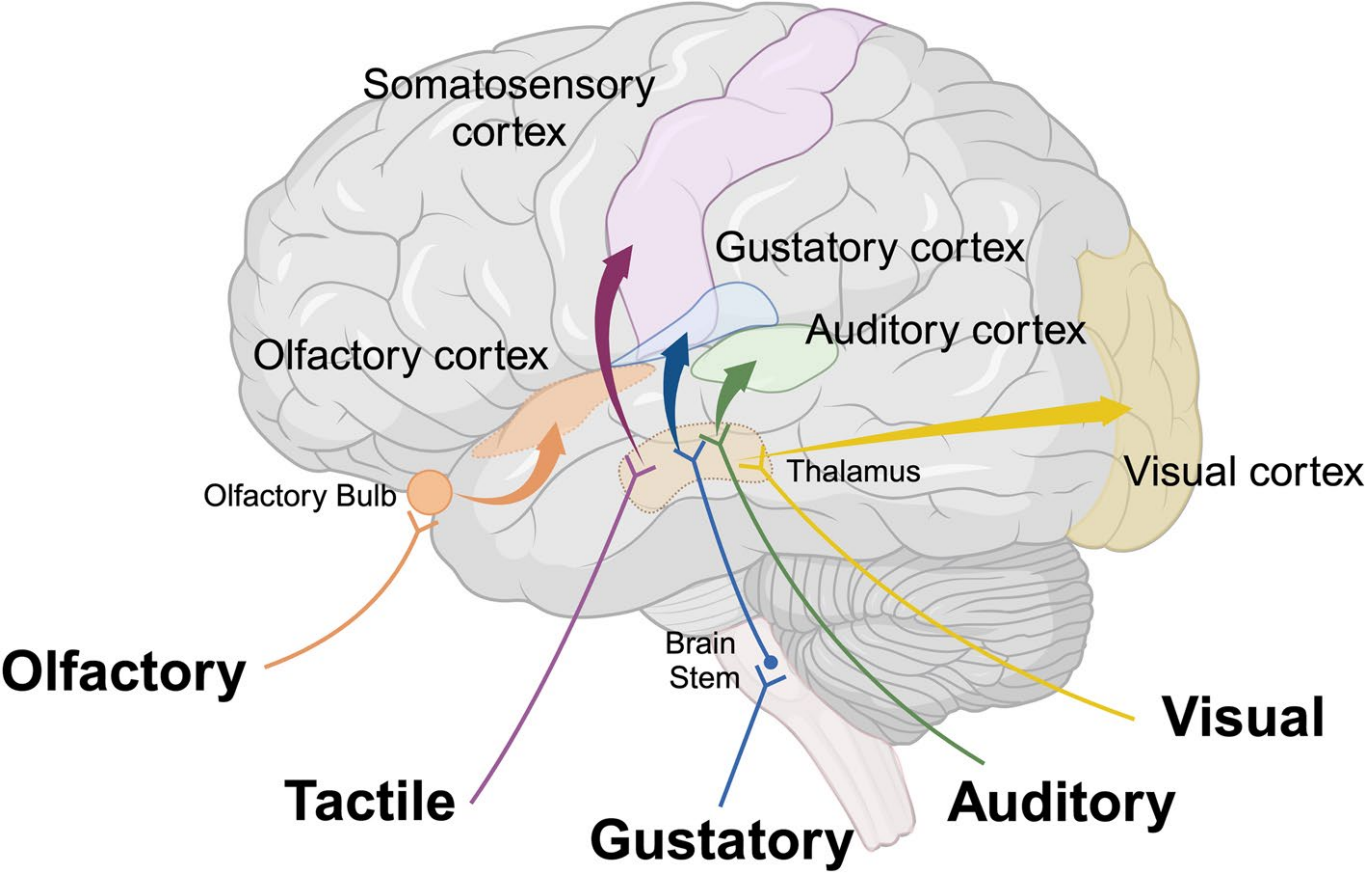
Neuroanatomical Regions of Sensory Information in the Cerebral Cortex. Multimodal regions facilitate the processing of information derived from diverse sensory areas within the brain. Image created using Biorender

Sensory loss may impact the ability of the cerebral cortex to generate perception in modalities such as vision, hearing, olfaction, and touch, extending beyond the effects of aging. Indeed, sensory deficits, including decreased visual contrast sensitivity [5], hearing loss [6], and olfaction deficits [7], are frequently observed in patients with AD. These observations have prompted extensive research into the association between sensory dysfunction and the pathology of AD. These deficits are intricately linked to alterations of synaptic plasticity and function, potentially precipitating cognitive impairments and suggesting that sensory impairments might not only be consequences of AD but could also contribute to its onset or progression [8] (Fig. 1). Aβ deposition, tau pathology, and neuronal loss within the neocortical regions of both primary and association cortices could potentially be the underlying causes of the sensory perception deficits observed in AD brains. The pathological changes in these areas display similarities across various sensory modalities. They are generally more pronounced in sensory association areas than in primary sensory cortices, particularly in the superficial cortical layers, where changes are most marked [9]. This differential pattern of impairment may explain the variability observed in sensory function: basic sensory functions such as visual acuity and auditory frequency discrimination are typically preserved, while more complex sensory functions, such as visual contrast sensitivity and auditory processing in noisy environments, tend to be compromised at early stages. These deficits, more evident in the sensory association areas and especially in the superficial layers, highlight the areas of the cortex that are vulnerable in AD [10–15].

**Fig. 2:**
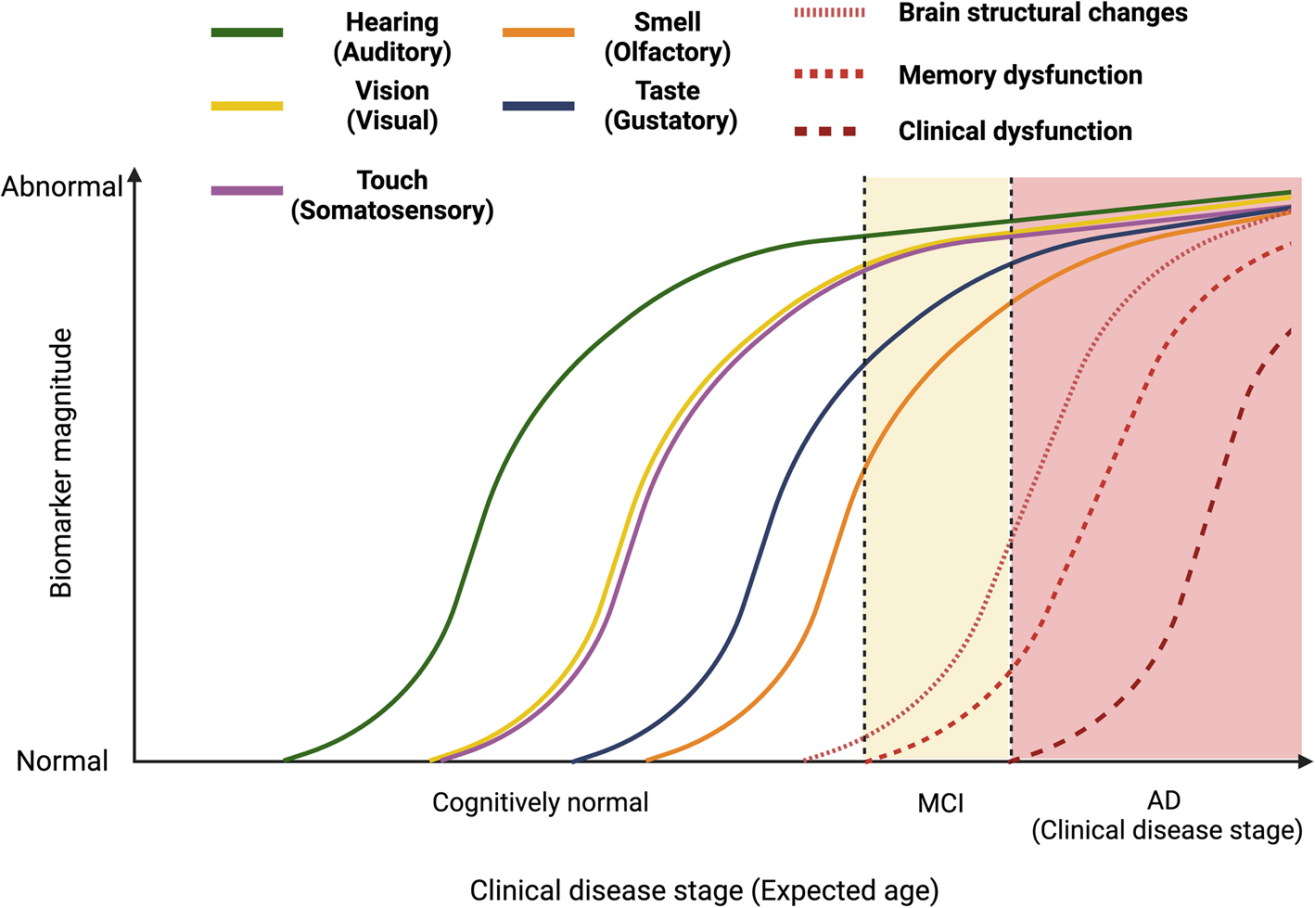
Hypothesized Development of Sensory Functional Impairment during the Pathological Progression of AD. This figure depicts the onset of sensory impairment alongside the emergence of other indicators within the context of the AD pathological cascade. MCI, mild cognitive impairment. Image created using Biorender

Research consistently indicates that sensory deficits can exacerbate brain vulnerability to neuropathological changes, including amyloid deposition, tau pathology, and neuronal loss, thereby increasing the risk of developing AD [7, 16–25] (Fig. 2). Understanding this relationship requires a shift in research and therapeutic strategies to address not only the cognitive symptoms of AD but also to consider sensory impairments as potential early indicators and contributing factors in the disease progression. Indeed, sensory stimulation therapies targeting these cortical regions have shown promise in reducing AD symptoms [19, 26–31]. However, understanding the intricate relationship between sensory impairments and AD progression remains an under-researched area. This review examines the intricate relationship between sensory impairments and AD, delving into the multifaceted nature of sensory decline in aging populations and its potential role as a harbinger or accelerator of AD. We aim to provide a nuanced understanding of how sensory dysfunctions, particularly in olfactory, auditory, and visual systems, not only serve as early indicators of AD but may also contribute to its pathogenesis, underscoring the need for an integrated approach in AD research and management.

## Main

### Auditory impairment

Age-related hearing loss (ARHL), or presbycusis, is a common sensory impairment in older populations. The prevalence of ARHL is often linked with the aging process and is highly prevalent among older adults. The incidence of hearing impairment shows a progressively increasing trend with age, as roughly 25% of individuals aged 60 years and over encounter disabling hearing loss [32]. Clinical hearing loss commonly initiates when bilateral hearing thresholds exceed 25 dB, beginning with higher frequencies and progressively extending to lower ones. Additionally, affected individuals frequently face challenges in hearing comprehension amidst background noise [33].

### The auditory system

The auditory system comprises two primary components: the peripheral hearing system and the central auditory system [34, 35]. The peripheral system includes the outer, middle, and inner ears, as well as the cochlear nerve, and is responsible for pivotal auditory functions such as sound detection [35]. Auditory information originating from the cochlea travels through a sequence of neural structures, including the superior olivary complex, lateral lemniscus, inferior colliculus, and medial geniculate nucleus, prior to arriving at the auditory cortex. The auditory cortex is located in the superior temporal gyrus of the temporal lobe and exhibits a precise tonotopic map that corresponds to cochlear frequencies [34]. It is comprised of discrete areas, namely AI (the foremost auditory cortex, Brodmann area 41) and AII (secondary area of auditory processing, spanning Brodmann area 42, anterior, ventral, ventral-posterior, and posterior auditory fields) [36]. AI plays a key role in processing the temporal features of complex auditory signals, including speech and music, sound localization, and identifying sources in auditory scene analysis [37–39]. The hippocampus detects new acoustic stimuli and inhibits duplicate auditory information [40]. Studies on animals show that neurons responsive to modulated tones and noise for frequency and amplitude are found in areas that are anterior and ventral to AI, while the areas posterior to AI contain neurons that have broader frequency tuning, longer tone response latencies, and lower following rates for acoustic frequency and amplitude modulations [41].

Acquired hearing loss mainly occurs due to cochlear damage, whilst AD is associated with cortical degeneration that typically starts in multimodal cortical regions. This raises the fundamental question of how these two ailments are linked. This query has theoretical importance, considering the existence of multiple potential biological and psychological pathways connecting peripheral auditory function with the widespread cortical changes associated with dementia. Moreover, studying the mechanisms underlying the link between hearing loss and cortical degeneration holds practical importance, due to the possibility of effective treatments for hearing loss with cochlear implants or hearing aids, unlike the difficult task of reversing cortical degradation.

### Auditory system dysfunction and AD

Several studies have suggested a potential link between AD and ARHL, which is considered a modifiable risk factor for AD dementia [42–44]. Studies also provide evidence that ARHL precedes the clinical onset of dementia by 5 to 10 years [45]. The initial report indicating the possible connection between hearing loss and cognitive impairment suggested that individuals with dementia were more likely to have hearing impairments than cognitively healthy older adults [46]. Since then, numerous investigations have shown an association between ARHL and the risk of developing cognitive decline or dementia. Furthermore, some studies have suggested that the use of hearing aids may delay or even prevent cognitive decline [47–50]. The Lancet International Commission on Dementia, Prevention, Intervention, and Care estimates that eliminating mid-life hearing loss could potentially reduce dementia risk by approximately 7% [23]. Recent research indicates a positive correlation between the severity of peripheral hearing loss, ranging from mild to severe, and the associated risk of dementia, with the risk increasing two to five times higher [51–53].

Studies have shown that hearing impairment is associated with higher levels of tau protein in the cerebrospinal fluid (CSF) and increased tau deposition in the brain. Specifically, poor hearing performance has been linked to elevated tau levels rather than Aβ deposition [54, 55]. This is particularly relevant in the context of hearing loss, as auditory processing deficits may exacerbate cognitive decline through increased neural activity and subsequent tau accumulation [56, 57]. Research indicates that higher tau PET signal correlates with regions of the brain involved in auditory processing and memory, such as the medial temporal lobe (MTL) [58–60]. These findings support the hypothesis that tau pathology in these regions is associated with both cognitive decline and auditory deficits in patients with AD. Also, it has been posited that auditory deficits may lead to modified neuronal activity within the MTL structures, potentially instigating or exacerbating the neuropathological processes of AD [61, 62]. The MTL structures, which are not typically linked to the auditory system, are emphasized here as they play a role in auditory processing, specifically in regard to analyzing sound patterns and memory functions [63, 64]. This may offer a new insight into the complex association between ARHL and the development of AD. Examining and testing the role of MTL in ARHL may considerably enhance our understanding of the fundamental mechanisms that unite these two phenomena.

#### Potential mechanisms linking hearing sysytem and AD

**Fig. 3:**
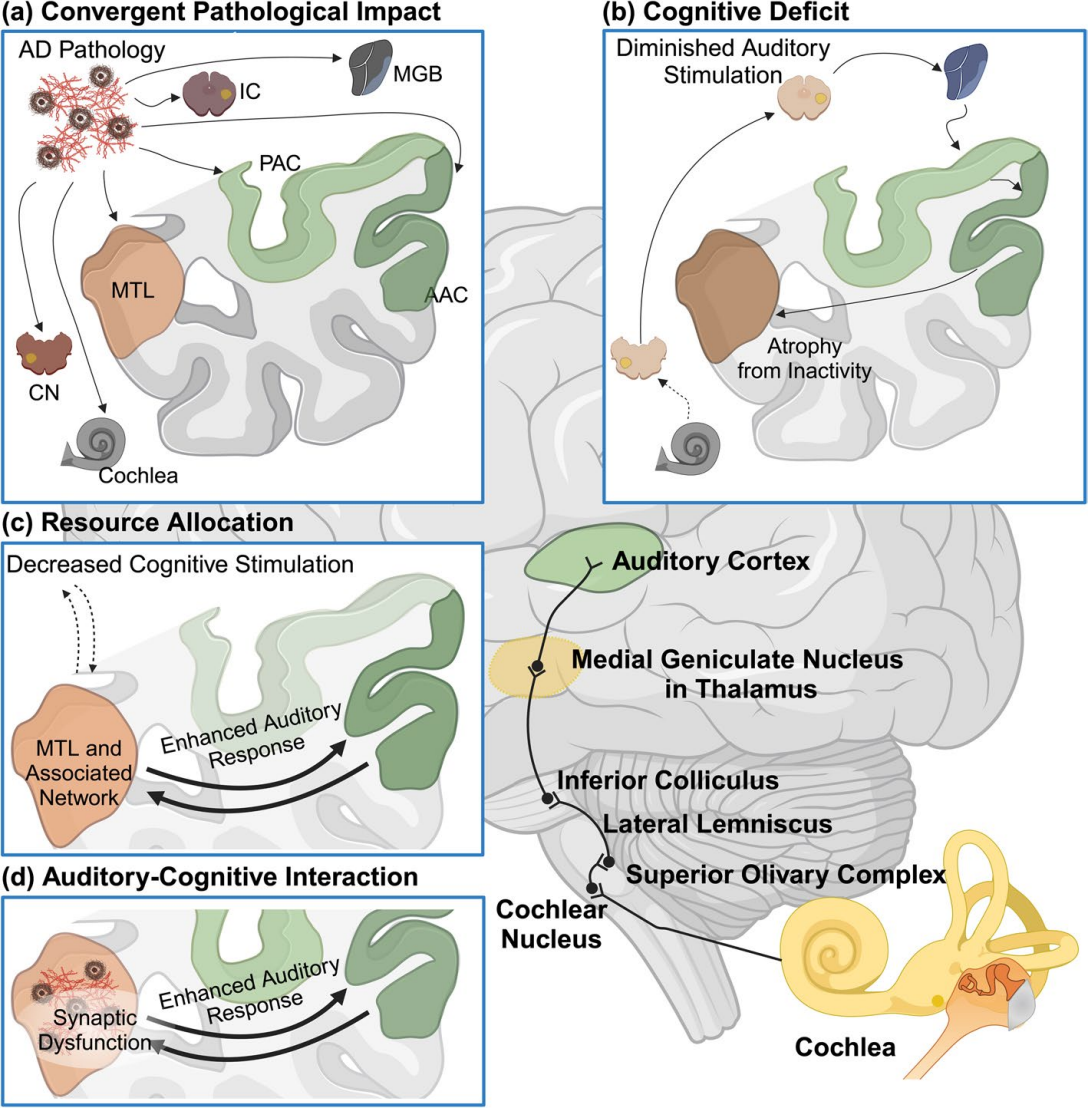
Potential Mechanisms Underlying the Association Between Hearing Impairment and AD. (a) Convergent Pathological Impact: Common pathological processes associated with AD or vascular disease impact both the cochlea and the ascending pathway, leading to hearing loss, while simultaneously affecting the MTL and causing dementia. (b) Cognitive Deficit: Hearing loss results in an impoverished cognitive environment, altering brain structure in the auditory cortex and hippocampus. This leads to decreased cognitive reserve and resilience against dementia due to a lack of cognitive stimulation. (c) Resource Allocation: Increased brain activity in the MTL and a broader network during speech-in-noise analysis competes for cognitive resources required for other higher cognitive functions. This mechanism is suggested to better explain cognitive deficits in older individuals with hearing loss rather than dementia itself. (d) Auditory-Cognitive Interaction: Hearing loss leads to increased activity related to pattern analysis in the MTL during challenging listening conditions, interacting with AD pathology. This model incorporates the same activity increase as in resource allocation mechanism but adds the interaction with the molecular bases of AD, specifically synaptic changes associated with the disease. Image created using Biorender

The precise pathological mechanisms responsible for ARHL are not yet fully elucidated. Nonetheless, it is probable that ARHL results from an amalgamation of acquired pathologies within variegated components of the complex auditory pathway (Fig. 3). Shared pathological processes in the cochlea, auditory pathways, and cortex have been implicated in the connection between hearing loss and dementia. It was suggested that high-frequency hearing loss is common and aligns with age-related cochlear degeneration, as opposed to AD-induced central pathway damage [65]. While current evidence underscores high-frequency hearing loss primarily originating from age-related cochlear changes, the direct attribution of such auditory deficits to central pathway damage by AD pathology is less established. Furthermore, despite the acknowledged role of vascular pathology as a contributory element to auditory function decline, the robust association between hearing loss and dementia endures, even after adjustments for vascular risk factors. This highlights the complexity of their relationship, suggesting that factors beyond vascular pathology may contribute a role in the linkage observed between hearing loss and cognitive decline [66].

Studies have shown that hearing impairment is associated with higher levels of tau protein in the cerebrospinal fluid (CSF) and increased tau deposition in the brain. Specifically, poor hearing performance has been linked to elevated tau levels rather than Aβ deposition [54, 55]. This is particularly relevant in the context of hearing loss, as auditory processing deficits may exacerbate cognitive decline through increased neural activity and subsequent tau accumulation [56, 57]. Research indicates that higher tau PET signal correlates with regions of the brain involved in auditory processing and memory, such as the medial temporal lobe (MTL) [58–60]. These findings support the hypothesis that tau pathology in these regions is associated with both cognitive decline and auditory deficits in patients with AD. Also, it has been posited that auditory deficits may lead to modified neuronal activity within the MTL structures, potentially instigating or exacerbating the neuropathological processes of AD [61, 62]. The MTL structures, which are not typically linked to the auditory system, are emphasized here as they play a role in auditory processing, specifically in regard to analyzing sound patterns and memory functions [63, 64]. This may offer a new insight into the complex association between ARHL and the development of AD. Examining and testing the role of MTL in ARHL may considerably enhance our understanding of the fundamental mechanisms that unite these two phenomena.

Hearing loss might result in decreased cognitive stimulation and a sensory-deprived environment due to diminished perception of speech and language which could elevate the risk of dementia. Numerous animal studies have demonstrated structural and behavioral changes in response to enriched environments, which may establish the cognitive reserve and serve as a protective factor against dementia [67–69]. Furthermore, impaired hearing can impact brain structure and function due to the reduction of verbal and emotional stimuli, and cognitive decline may result from poor social interactions [70–74]. Moreover, Hearing loss may increase cognitive load during auditory processing, reallocating cognitive resources from other functions such as attention and working memory [75–77]. This cognitive resource reallocation may precede the clinical manifestation of AD, with auditory cognitive networks experiencing diminished stimulation. Specifically, the pathology involves neuroplastic changes and alterations in brain structure arising from decreased sensory stimulation—factors that may precede and subsequently potentiate the risk of AD [78–80]. These neuroplastic changes differ from the alterations in brain activity observed during the progression of AD, which are posited to contribute directly to cognitive deficits. Such impairments may develop independently of the specific molecular and neuronal pathologies characteristic of AD [81], suggesting that the etiology of cognitive decline in the context of hearing loss is multifactorial and extends beyond AD-related neurodegeneration.

#### Hearing loss and dementia: significance and future directions

Hearing impairment has been strongly correlated with an increased likelihood of mild cognitive impairment (MCI) and accelerated cognitive degeneration. Specifically, those with impaired hearing face a markedly higher risk of developing MCI than individuals with normal hearing levels. Furthermore, evidence suggests that using hearing aids can diminish this risk, implying a protective effect against cognitive decline [82]. The use of hearing aids has been linked to a substantial reduction in the risk of cognitive decline. A study published in The Lancet found that hearing aid use can reduce the risk of cognitive decline by nearly half in older adults at high risk for dementia [83]. Another large-scale study involving over half a million adults indicated that hearing aids might prevent or delay the onset and progression of dementia [84]. Studies have demonstrated that individuals using hearing aids experience a slower rate of cognitive decline compared to those who do not use them. Ongoing research and clinical trials continue to explore the relationship between hearing loss and cognitive decline. For example, randomized controlled trials are investigating whether hearing rehabilitation can improve cognitive function in older adults with hearing impairment and MCI [85]. While auditory impairment alone may not be the most sensitive or specific diagnostic marker for AD, addressing hearing loss through contemporary interventions such as hearing aids holds promising potential in mitigating cognitive decline and delaying the progression of AD. Continued research and early intervention strategies are essential to fully understand and leverage the benefits of hearing rehabilitation in the context of AD.

## Olfactory impairment

Olfactory perception is a complex sensory process that results from the integrated function of olfactory receptors, the olfactory nerve, and various central nervous system structures. The confluence of these myriad factors collectively underscores the intricacy of age-related olfactory loss and its profound impact on the aging population. Remarkably, a substantial proportion of affected individuals may persist in a state of unawareness regarding their compromised olfactory faculties [86]. In the elderly population, olfactory dysfunction prevalence surpasses 50% among individuals aged 65 to 80, escalating to 80% in those over the age of 80. This underscores the pervasive nature of olfactory issues within this demographic [87]. Profound olfactory dysfunction substantially detracts from the quality of life, as demonstrated by empirical findings [88]. While some olfactory deficits arise from congenital or idiopathic conditions, it is important to recognize that most olfactory dysfunction in adults is attributable to allergies, nasal polyps, smoking, and prior history of head or facial trauma [89]. These conditions often fall under conductive (transport-related) or sensorineural categories, each affecting olfaction in different ways. Before concluding that changes in olfaction are indicative of neurodegenerative diseases such as AD and Parkinson’s Disease (PD), it is essential to rule out these more common causes. Nevertheless, once these factors are excluded, there remains a notable correlation between olfactory dysfunction and neurodegenerative diseases [90]. The involvement of brain regions integral to olfactory processing, notably the olfactory bulb and entorhinal cortex, in the initial stages of neuropathological processes in AD, underscores the potential of olfactory dysfunction as a promising early biomarker for the disease [91]. Interestingly, olfactory training has been associated with augmented cortical thickness in the hippocampus among patients with MCI, notwithstanding the absence of notable alterations in olfactory bulb volume. These observations posit olfactory training as a potential early intervention capable of mitigating hippocampal atrophy [92]. Consequently, the burgeoning interest in olfactory deficits within the domain of AD has become increasingly evident in both basic scientific and clinical research, underscoring the importance of studying olfactory dysfunction in the context of AD. This focus on both the foundational understanding of the disease and its practical clinical implications may, in turn, offer valuable insights into the early detection and monitoring of this neurodegenerative condition.

### The olfactory system

**Fig. 4:**
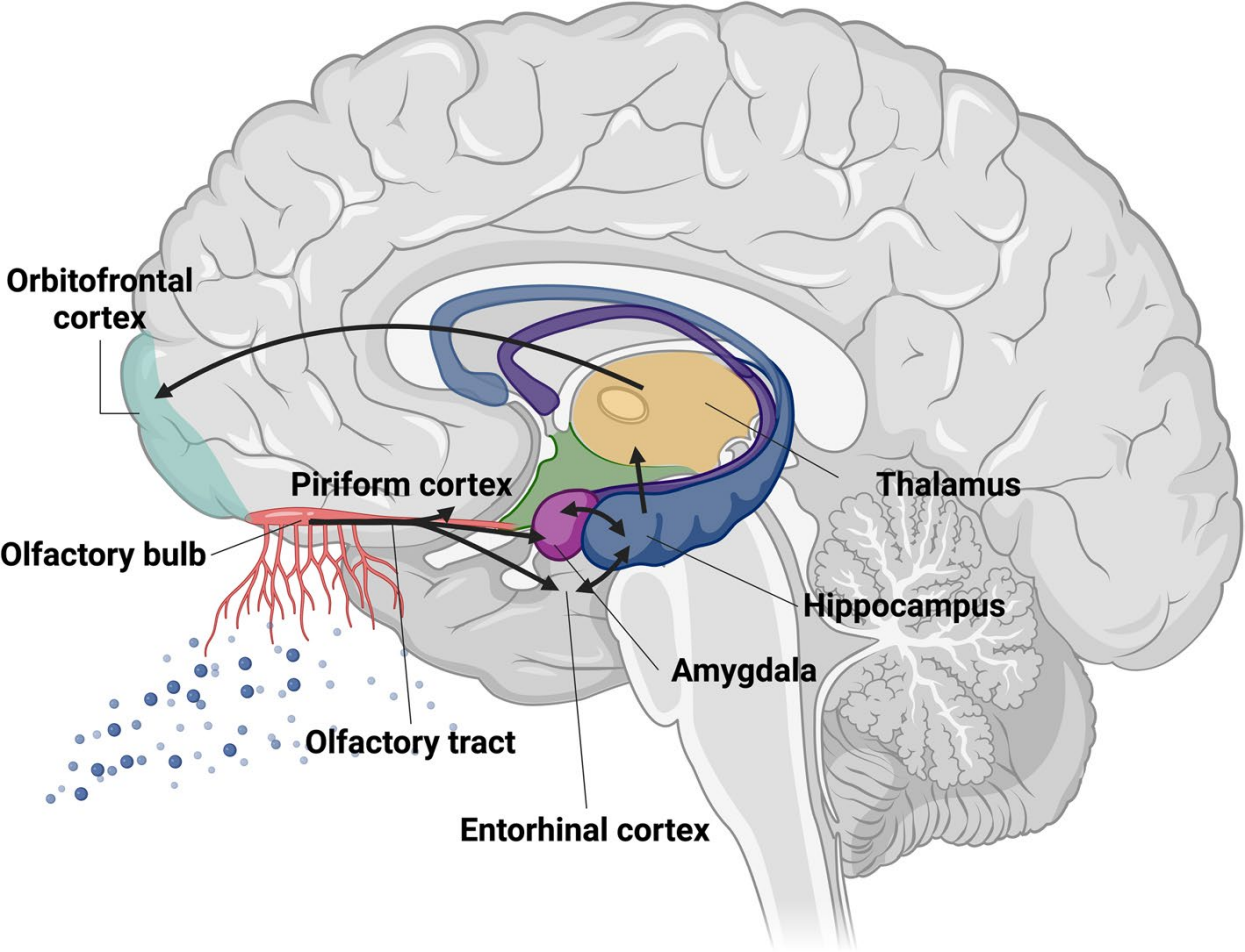
Critical Brain Regions Involved in Olfactory Network. Scheme of the olfactory system detailing the process of olfaction. Olfactory sensory neurons transduce odor information through electrical signals, initiating neurotransmitter release in the olfactory bulb. Regions implicated in early olfactory processing encompass the olfactory bulb and tract, the piriform cortex, amygdala, entorhinal cortex, and hippocampus. Image created using Biorender

Specialized olfactory nerves, exquisitely adapted to the olfactory sense, are located within the olfactory epithelium. This distinctive lining occupies the upper nasal cavity and assumes a pivotal role in shaping our olfactory perception. In mammals, odor signals processing commences in the olfactory epithelium, wherein olfactory sensory neurons function as the primary neural entities. The olfactory system orchestrates the transmission of chemical signals from the sensory epithelium and bulb to the olfactory cortex through a sequential synaptic interface [93–95]. Functioning as a pivotal convergence site, the olfactory bulb amalgamates the peripheral olfactory system with the central subcortical systems, facilitating the interconnection between the axons of olfactory sensory neurons and the dendrites of mitral cells. Comprising the anterior olfactory nucleus, olfactory tubercle, piriform cortex, entorhinal cortex, and amygdala, including the orbitofrontal regions, the primary olfactory cortex represents a comprehensive neural network. This network further extends to project neural signals towards the secondary olfactory cortex in the orbitofrontal cortex (Fig. 4) [96]. Afferent input originating in the olfactory bulb is transmitted to the primary olfactory cortex via the olfactory tubercles, encompassing axons derived from mitral/tuft cells and GABAergic interneurons [97]. These cells undergo differentiation from progenitor cells migrating from the subventricular zone [97], and the pathway constitutes the inaugural cranial nerve within the central nervous system. Several investigations have consistently reported the existence of neuropathological alterations concomitant with olfactory disorders within the context of neurodegenerative diseases. These changes may impact diverse anatomical components, including the olfactory epithelium, olfactory bulb/tract, primary olfactory pathways, and their secondary targets−specific cerebral regions that receive input from the primary olfactory pathways [98]. Notably, brain regions intricately involved in olfactory processing, such as the olfactory bulb and entorhinal cortex, demonstrate early and pronounced neuropathological changes in AD [99–101]. This observation posits olfactory function as a promising candidate for serving as a potential biomarker for AD.

### The olfactory system and AD

The olfactory system in early AD detection has attracted attention due to observations of early neuropathological changes in areas critical to olfaction, such as the olfactory bulb and entorhinal cortex. These early changes suggest that olfactory dysfunction could serve as an early indicator of AD, potentially manifesting before recognizable cognitive deficits become apparent [102–104]. As a result, research into the olfactory system has expanded, with studies examining its implications in AD pathology and its potential utility in early diagnosis [101, 105].

A decline in olfactory capabilities is often observed concurrently with the deterioration of visuospatial cognitive functions, an early symptom of dementia [106]. This concurrent deterioration is likely linked to neurodegeneration in areas such as the parahippocampal gyrus (PHG) and orbitofrontal cortex (OFC), which are responsible for integrating sensory information and are among the first regions to exhibit atrophy in the progression of AD, indicating a potential shared pathway for the decline in both sensory and cognitive domains [107, 108]. Research has shown that lesions in the PHG or its rodent equivalent, the postrhinal cortex (POR), result in significant visuospatial learning and memory deficits, which affect memory retrieval more than encoding [109]. The PHG is involved in contextual processing, which is important for understanding and navigating spatial environments [110]. Additionally, the uncinate fasciculus, a pathway connecting the anterior temporal lobes (including the PHG) to the OFC, plays a role in visuospatial memory, and its disruption has been linked to visuospatial deficits in AD. Therefore, neurodegeneration in these regions likely impairs the circuits responsible for visuospatial processing, contributing to the observed cognitive decline in visuospatial functions in early dementia.

The olfactory test introduced by Richard L. Doty in 1984 has become a mainstay in clinical research and diagnosis [111], with numerous studies confirming severe olfactory loss in patients with AD and Parkinson Disease, especially challenges in odor identification [112–116]. Odor identification tests are not currently used as standard diagnostic markers for AD in clinical practice. However, research has identified them as significant predictive markers for AD progression, with studies showing that individuals with intact olfactory function may have a lower risk of developing dementia [117]. The studies collectively suggest that olfactory dysfunction may serve as an early indicator of AD-related neurodegeneration and is closely associated with tau accumulation in specific brain regions. Lower olfactory identification scores were associated with a higher risk of developing MCI due to AD, and odor identification deficits are predominantly linked to tau accumulation in key olfactory pathway areas [118–120]. Specifically, lower the university of Pennsylvania smell identification test (UPSIT) scores correlate with increased tau deposition in the medial temporal cortex, hippocampus, middle and inferior temporal gyri, inferior parietal cortex, and posterior cingulate [121]. A meta-analysis found that tau PET imaging might be more strongly associated with olfactory impairment than amyloid PET, emphasizing the potential of combining olfactory tests with other biomarkers for better prediction of cognitive decline.

While olfactory dysfunction is often overlooked in clinical practice, it has been linked to an increased likelihood of AD dementia and higher mortality rates [122–125]. Recent findings link olfactory impairments to various neurological conditions, highlighting the broader neurological importance of the olfactory system [126–128].

The underreporting of olfactory dysfunction is notable; while only 6% of patients with AD report a loss of smell, objective measures reveal significant impairment in up to 90% of cases [129]. This discrepancy highlights the critical vulnerability of individuals with undetected olfactory impairments to the progression of AD, particularly when coupled with anosognosia, suggesting the need for heightened clinical attention to olfactory testing in dementia screening.

#### Potential mechanisms linking olfactory system and AD

Recent research has shed light on potential mechanisms linking the olfactory system to AD, focusing on the accumulation of Aβ peptides and its impact on olfactory dysfunction. Studies using animal models have shown that Aβ peptides accumulate in specific regions of the olfactory system, such as the olfactory epithelium (OE) and olfactory bulb (OB). This accumulation is associated with early olfactory dysfunction in AD, characterized by partial olfactory dysfunction or hyposmia. The olfactory sensory neurons (OSNs) in these regions are particularly affected, leading to disruptions in their turnover and function [130, 131]. It appears that the physiological organization of the olfactory epithelium and bulb, which is conserved across species, is integral to the neurodegenerative processes observed in the olfactory pathways of AD. The degeneration of olfactory pathways, including the OE and OB, contributes significantly to olfactory dysfunction in AD, marking a shift from previous assumptions that higher cortical deficits were the primary cause of olfactory impairments in AD. Emerging insights point to olfactory system neuropathology and neurodegeneration as the primary contributors to olfactory dysfunction in AD [104, 132–134]. Odors serve as potent memory cues, and the connection between the olfactory system and the hippocampus is important for episodic memory, which often declines first in AD. Proficiency in odor identification reflects the depth of semantic information and naming ability, suggesting that identification deficits could indicate wider cognitive dysfunction, including hippocampal degeneration [135, 136].

The pathological processes involved in the amyloidogenic metabolism of amyloid precursor protein (APP) and the resulting neuroinflammatory reactions within the olfactory pathways are key contributors to olfactory dysfunction in AD. These processes lead to the generation and accumulation of toxic Aβ oligomers, which can cause synaptic toxicity and cellular damage [137]. The correlation between cognitive function and oligomerized Aβ proteins in nasal discharge highlights the potential research impact of nasal-fluid biomarkers in understanding AD pathology [138]. This is similar to the use of CSF and plasma biomarkers, providing a non-invasive method to detect AD-related changes early in the disease progression [139]. There is also a hypothesis that external pathogens might exploit the olfactory pathway to initiate neuroinflammation, which could play a role in the pathogenesis of AD [101]. The olfactory system provides a direct anatomical connection to the brain, making it a plausible route for pathogen entry [133]. These insights offer new avenues for early diagnosis and a deeper understanding of AD progression through the lens of olfactory dysfunction.

#### Olfactory loss and dementia: significance and future directions

Olfactory impairment is increasingly acknowledged as a promising non-invasive biomarker for AD, particularly through the use of odor identification tasks. Despite this, the integration of olfactory measurement with verbal skills and cultural context may confound its application as a diagnostic tool [140]. Olfactory identification is intricately linked to higher cognitive functions and shows a significant decline with age, more so than odor detection thresholds, implicating both sensory and cognitive aspects of the olfaction [141, 142]. Therefore, understanding olfactory tasks, such as discrimination and identification, is pivotal in diagnosing AD. Discrimination involves the sensory ability to differentiate between various odors, a fundamental test of the olfactory system sensitivity. Identification, however, requires the integration of cognitive processes (memory and higher-order thinking) to recognize and name odors. This distinction is pivotal not only in understanding the specificity of olfactory deficits as risk factors for AD but also in their correlation with the progression and severity of the disease [98]. Future research endeavors should strive to characterize these olfactory deficits with more nuanced perceptual and cognitive details, thereby enriching our understanding of the associated pathophysiology. This should encompass in vivo assessments focused on the analysis of Aβ, tau, and alpha-synuclein proteins in individuals exhibiting olfactory dysfunction. Given the prevalence of overlapping neuropathologies in AD and other neurodegenerative diseases, an integrated approach to research, intertwining olfactory impairment with neuropathological markers, is imperative for advancing our understanding of these complex disorders.

The UPSIT is a widely used 40-item smell identification test. Studies have shown that a 10-item subset of the UPSIT can achieve a sensitivity of 88% and a specificity of 71% for identifying AD. For identifying an amnestic disorder, the sensitivity is 74% and specificity is 71% [143]. The test has been validated as a useful screening tool for AD-related amnestic disorders, with sensitivity and specificity comparable to other established biomarkers. The Brief Smell Identification Test (BSIT), a shorter version of the UPSIT, has been proposed for primary care settings due to its quick administration time and cost-effectiveness. It has shown similar effectiveness to the UPSIT in distinguishing and predicting MCI and AD dementia [144, 145]. However, implementing olfactory assessments like the UPSIT in clinical settings presents considerable challenges. These tests are time-consuming, and demand sustained attention and intact cognitive functions, which are frequently compromised in patients with dementia or cognitive impairments. Additional obstacles include physical limitations, environmental distractions, and the influence of cultural or educational differences on odor recognition. Moreover, deploying these tests in practice requires substantial resources such as specialized training, test kits, and dedicated spaces [146, 147]. These factors collectively limit the use of olfactory assessments primarily to research environments rather than routine clinical application. Despite these challenges, the potential of olfactory testing in AD diagnosis and progression monitoring remains meaningful. In clinical trials, odor identification tests have demonstrated similar sensitivity and specificity to CSF biomarkers for detecting progression within the AD spectrum, from amnestic MCI due to AD through to more advanced stages of AD dementia [45, 148]. Cross-sectional and longitudinal population-based studies have further elucidated the association of olfactory identification deficits with impairments in memory and executive functions [149–151]. However, in clinical practice and broad population-level screening, the future of AD diagnosis will likely incorporate blood-based biomarkers. Recent advancements have shown that plasma biomarkers, such as Aβ_42_ and phosphorylated tau (pTau217), are reliable indicators of AD pathology, offering advantages such as being less invasive, cost-effective, and time-efficient [152–155].

## Visual impairment

As the global population ages, the prevalence of visual impairment escalates, presenting considerable challenges to healthcare systems and affects the quality of life for older adults [156]. Epidemiological studies have identified refractive errors, age-related macular degeneration, cataracts, and glaucoma as the leading causes of this condition [157]. Notably, refractive errors, which are readily correctable with glasses or contact lenses, account for a substantial portion of visual impairment cases [158]. This fact underscores the potential for reducing the burden of visual impairment through enhanced access to corrective solutions. The incidence of visual impairment notably increases with age, affecting an estimated 20–22% of individuals aged 70 and above [159]. Projections indicate that the global prevalence of moderate to severe visual impairment is set to rise from 217 million cases in 2015 to approximately 588 million by 2050 [159, 160]. This burgeoning demographic is associated with heightened risks of hospitalization and augmented healthcare costs, underscoring the pressing need for comprehensive care strategies [161, 162].

Pathophysiological changes within the aging eye contribute to visual impairment. The lens becomes denser and less transparent, leading to cataracts, while degenerative changes in the retina, as seen in age-related macular degeneration, directly impair vision [163]. Glaucoma, characterized by increased intraocular pressure, can lead to optic nerve damage and visual field loss, illustrating the complex interplay of age-related changes that compromise visual function [164]. Moreover, these functional limitations are compounded by psychological challenges, as individuals with visual impairment are at an increased risk of experiencing depression and anxiety, exacerbating the overall impact on their well-being [165]. Recent insights from the American Geriatrics Society and the National Institute on Aging have emphasized the frequent co-occurrence of cognitive and sensory impairments, particularly in vision and hearing [166]. In conclusion, addressing visual impairments holds promising potential for enhancing cognitive function.

### The visual system

**Fig. 5:**
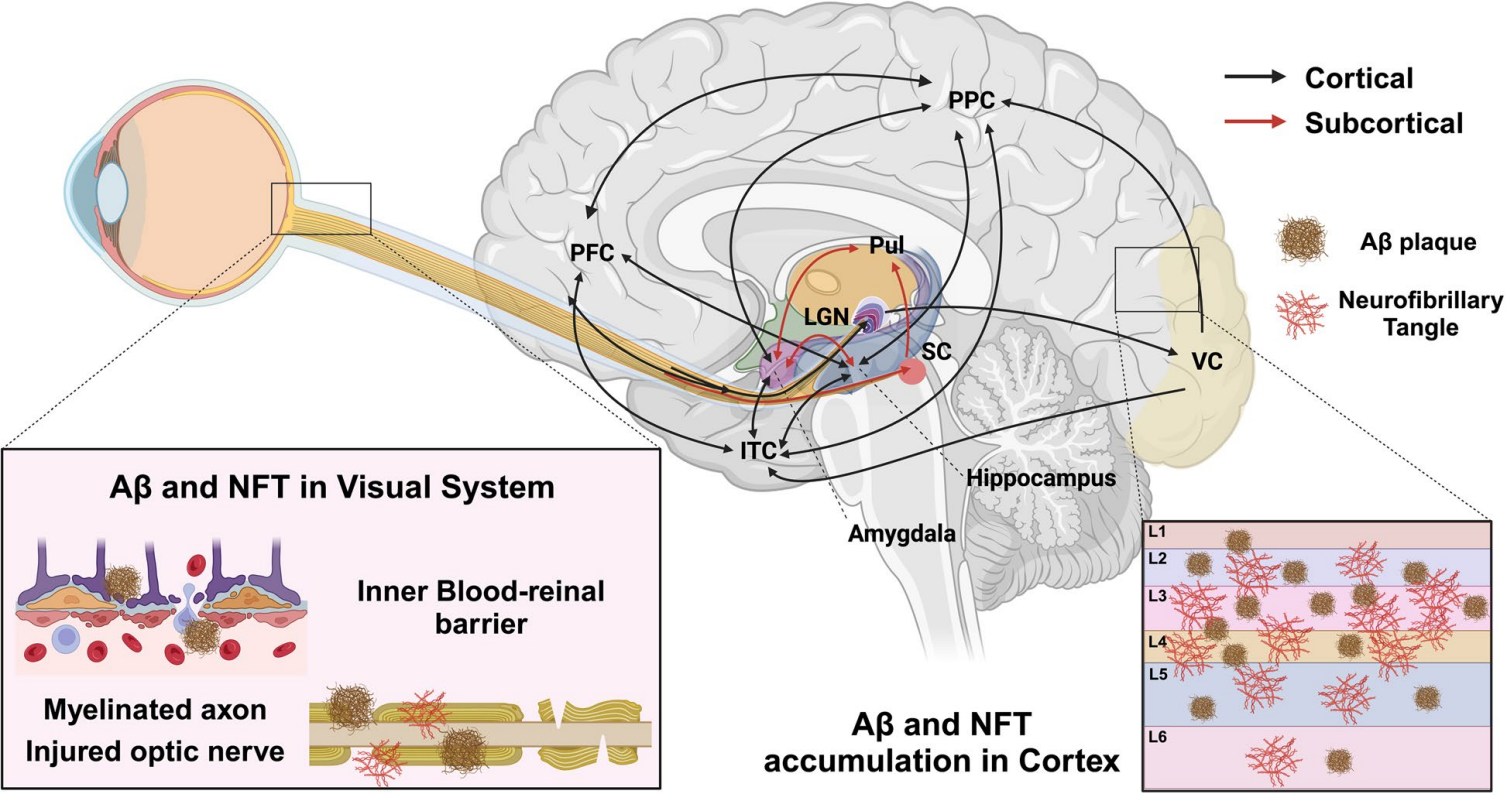
Impact of Visual System on AD Progression. This visual system illustration delineates discernible alterations observed in individuals affected by AD. Pathological hallmarks of AD, specifically amyloid plaques and tau tangles, manifest prominently in pivotal brain regions associated with visual function, including the visual cortex, pulvinar nucleus, lateral geniculate nucleus, suprachiasmatic nucleus, and superior colliculus. These pathological changes intricately correlate with disruptions in their associated functions. Within the ocular domain, atients with AD exhibit optic nerve degeneration, marked by the loss of axonal projections. Furthermore, AD is concomitant with a reduction in retinal thickness and a decline in retinal vasculature. ITC: inferior temporal cortex; LGN: lateral geniculate nuclei; PFC: prefrontal cortex; PPC: posterior parietal cortex; Pul: pulvinar; SC: superior colliculus; VC: visual cortex. Image created using Biorender

The visual system, a complex network integral to the reception and processing of visual stimuli, operates through afferent and efferent pathways. The afferent pathway begins in the retina, structured with specialized neuronal layers communicating through synapses, functioning as an extension of the central nervous system (CNS). Photoreceptor cells in the outer layer of the retina capture incoming light, initiating a neural signal cascade that culminates at the retinal ganglion cells (RGCs), whose axons form the optic nerve (ON). These axons project to key CNS structures such as the lateral geniculate nucleus (LGN) in the thalamus and the superior colliculus (SC) in the midbrain [167] (Fig. 5). The LGN acts as a vital hub, relaying signals to the primary visual cortex, which, in collaboration with higher-order brain regions, undertakes complex tasks like object recognition and spatial processing [168]. Simultaneously, the efferent visual pathway involves the frontal eye field, parietal eye field, and basal ganglia, orchestrating saccades—rapid, precise movements between fixation points. The signals for these movements are modulated by the superior colliculus and directed by the saccade burst generator in the brainstem, with the dorsolateral prefrontal cortex playing a key role in their voluntary control [169, 170].

Current research explores the retina as a potential conduit to brain health, investigating how ocular examinations could deepen understanding of CNS disorders. The process of light transduction into neural signals by the retina offers a unique opportunity to observe CNS health [171]. The transmission of these signals from the photoreceptors, through the RGCs, to the visual processing centers in the brain reflects a dynamic interplay between sensory input and neural processing. This pathway facilitates not just our perception of the world but may also hold keys to early detection and monitoring of CNS pathologies, including neurodegenerative diseases. Measurements of retinal nerve fiber layer thickness and macular volume are not only indicators of retinal health but may also signal neurodegenerative conditions. A comprehensive understanding of retinal structure and function is thus essential for advancing ocular assessments in the context of neurological evaluation and could lead to novel diagnostic and therapeutic approaches for neurodegenerative diseases [172–175].

### The visual system and AD

In the past few decades, considerable research attention has been directed towards elucidating the ocular manifestations associated with AD [176–179]. A seminal case-control study, focusing on the optic nerves of patients with AD compared to healthy controls, provided robust evidence for RGC atrophy in AD [177]. In many instances, ocular symptoms have been observed to precede cerebral manifestations, suggesting that comprehensive eye examinations could serve as a valuable tool for early diagnosis of underlying neurological conditions [171]. Given the anatomical and functional connection between the eye and the brain, exploring early ocular manifestations in AD is becoming an important research avenue. These studies revealed that, compared to non-AD individuals, patients with AD exhibited constricted retinal veins, reduced retinal blood flow and RGC count, and thinner RNFLs, correlating these anomalies with retinal dysfunction [172, 180–182]. Significantly, a reduction in macular volume among patients with AD was found to correlate with performance on cognitive tests [183]. Prior research suggested that ocular degeneration in AD predominantly occurs in the posterior segments of the optic nerve, near the optic chiasm, indicating that while ocular symptoms are noticeable, the primary site of damage might be intracranial [184]. Furthermore, pathological accumulations of Aβ and p-tau have been identified in the lenses of patients with AD [185], and in the retina and retinal ganglion cells of transgenic AD mouse models [176, 186–190]. Similar to their effects in the brain, Aβ and p-tau in the eye are associated with various forms of ocular damage, including cataract formation, loss of retinal neurons, RNFL thinning, and impaired axonal development [179, 185, 186, 188].

Considering these findings, recent longitudinal cohort studies have illuminated the potential impact of treating visual impairment on future dementia risk. Cataract treatment, in particular, has been associated with a reduced risk of developing dementia including AD dementia, signifying the broader implications of addressing visual impairments. This contrasts with the results for glaucoma surgery, which does not typically lead to improved vision and has not shown a similar reduction in dementia risk [191]. The association between visual impairment and dementia risk is further supported by epidemiological research, which suggests that visual impairment may serve as a marker for short- and medium-term dementia risk [192].

In conclusion, the accumulating body of research underscores the intricate link between visual impairment and the risk of dementia, including AD. The ocular manifestations observed in patients with AD, particularly the changes in retinal structure and function, suggest that the eye could be a window to early detection and intervention in neurodegenerative diseases. As the global population ages, these findings underscore the importance of regular comprehensive eye examinations and the potential benefits of treating visual impairments in mitigating the burden of dementia.

#### Potential mechanisms linking visual system and AD

The interplay between visual impairment and the risk of dementia, including AD, has gained attention in recent years. The potential mechanisms linking the two conditions, while not fully understood, are believed to involve a variety of indirect pathways. One such pathway is the structural modification of the brain regions associated with vision. Research suggests that ocular impairments leading to reduced visual input can induce changes in and around the primary visual cortex. These changes can sometimes be reversed with appropriate ocular treatments, such as cataract surgery, indicating the plasticity of the brain in response to sensory input [193]. Enhanced sensory input can stimulate neuroplasticity, leading to structural improvements in brain regions affected by visual impairment [194]. This includes increases in grey matter volume and improvements in brain function in areas related to vision and cognition [195, 196]. Recent studies have shown that even functional brain networks far beyond the lesion site can be reorganized, further supporting the potential for visual recovery through neuroplasticity [197]. For instance, a study found that cataract surgery was associated with a slower decline in episodic memory, a key cognitive function, suggesting that improving visual function can positively impact cognitive health in individuals with AD [198]. The Fujiwara-kyo Eye Study found that elderly individuals who underwent cataract surgery had a significantly lower risk of developing MCI, suggesting that improving visual acuity can have broader cognitive benefits [199].

Another intriguing concept is cognitive resilience, which refers to the ability of an individual to maintain cognitive function despite the presence of extensive brain pathology, such as that seen in AD [200]. This concept is important because it helps explain why some individuals can sustain high levels of cognitive function even when their brains exhibit extensive AD-related damage. Consequently, interventions that improve ocular health, such as cataract surgery, might offer the most benefit to those with less innate resilience, potentially reducing their risk of developing dementia. Furthermore, engagement in visual-based leisure activities has been associated with enhanced cognitive resilience and a lower risk of dementia. Cohort studies have provided evidence that active participation in visually stimulating activities can contribute to cognitive health [200]. Better vision reduces the cognitive effort required to process visual information, allowing cognitive resources to be allocated more efficiently. This can lead to improved cognitive function and a lower risk of dementia, as individuals are more likely to engage in cognitively stimulating activities and maintain social interactions [201, 202]. These activities may help maintain cognitive function by promoting neuroplasticity and cognitive reserve.

#### Visual impairment and dementia: significance and future directions

Visual acuity, while often considered a straightforward measure of ocular health, can be influenced by various factors beyond mere optical clarity [203, 204]. It entails a complex interplay of sensory input and cognitive processing, similar to the way olfactory identification requires memory and higher-order thinking. The decline in visual function, more pronounced with age, implicates both the sensory apparatus and the cognitive domains of the brain. It is this decline that recent research suggests might be an early compensatory response to neural changes in AD, potentially offering a window for early therapeutic intervention [205]. Visual impairment is being increasingly recognized as an early indicator in AD research. Notable findings suggest that changes in visual acuity and related electrophysiological responses may precede the cognitive symptoms traditionally associated with AD, thus presenting an opportunity for early detection [206]. Furthermore, recent studies indicate that visual assessments, particularly those that measure changes in acuity and electrophysiological activity, could be integrated into routine screenings for AD [207]. Eye movement studies, for example, offer promising avenues for early detection and monitoring of AD by analyzing metrics such as saccadic movements, fixation patterns, and smooth pursuit [208–210]. These non-invasive techniques could complement existing diagnostic tools, providing a more comprehensive approach to AD detection and management.

An intriguing observation is the reported increase in visual acuity in some individuals at high risk for AD [211]. This contrasts with earlier research suggesting a decline in visual function in similar cohorts [212–216]. These findings require further investigation to determine whether it indicates an early neural response at the onset of AD or if it is due to hyperactivity in retinal cells caused by Aβ accumulation. Understanding the link between initial ocular signs and the progression of AD is a key focus area. There is a need to investigate the reasons behind altered visual acuity and to ascertain whether these changes are indeed precipitated by the impact of Aβ on the retina or reflect a general increase in neural adaptability.

In clinical settings, evaluating visual function has shown potential as a non-invasive method that may complement CSF biomarkers in detecting the progression from MCI to AD in some individuals. Further studies should investigate the reasons behind altered visual acuity in individuals at high risk for AD. Visual assessments that measure changes in acuity and electrophysiological activity should be integrated into routine screenings for AD to enhance early detection and intervention strategies. Research into the optic nerve and its connections to the brain should be expanded to understand how changes in the visual system might mirror brain health in AD. Integrating these various approaches (visual acuity assessments, electrophysiological measurements, and eye movement studies) can provide a more holistic understanding of how visual impairments relate to AD. By addressing these areas, researchers can develop innovative strategies to mitigate the burden of AD-related dementia and improve the quality of life for older adults.

## Gustatory impairment

The phenomenon of diminished taste, or hypogeusia, is a recognized yet understudied condition prevalent among the elderly, often not leading to significant clinical concerns. However, the influence of taste disorders on overall well-being and the ability to perform job-related tasks can be profound, particularly in severe cases where nutritional status and health may be compromised. Gustatory dysfunctions, reported with a prevalence of around 5% in the general population [217], are less frequently documented than olfactory disorders. Nonetheless, the clinical importance of gustatory disturbances warrants attention due to their potential impact on quality of life.

There is a notable lack of detailed research focusing specifically on taste disorders, which may be attributed to the complexity of the relationship between the gustatory system and other sensory modalities, including olfaction, somatosensation, and nociception. The challenge lies in isolating the specific contributions of each sensory system to the overall perception of flavor, a task that complicates research methodologies. The sensory experience of flavor is a result of the intricate integration of gustatory and olfactory stimuli, with concurrent neuronal activations observed in regions such as the insula, amygdala, and orbitofrontal cortex [218]. These areas are critical in the neural circuitry of flavor perception, highlighting the multifaceted nature of how flavor is processed and perceived in the human brain. Understanding these mechanisms is necessary for advancing the diagnosis and treatment of taste disorders, thereby enhancing patient care and quality of life in the aging population.

### The gustatory system

**Fig. 6:**
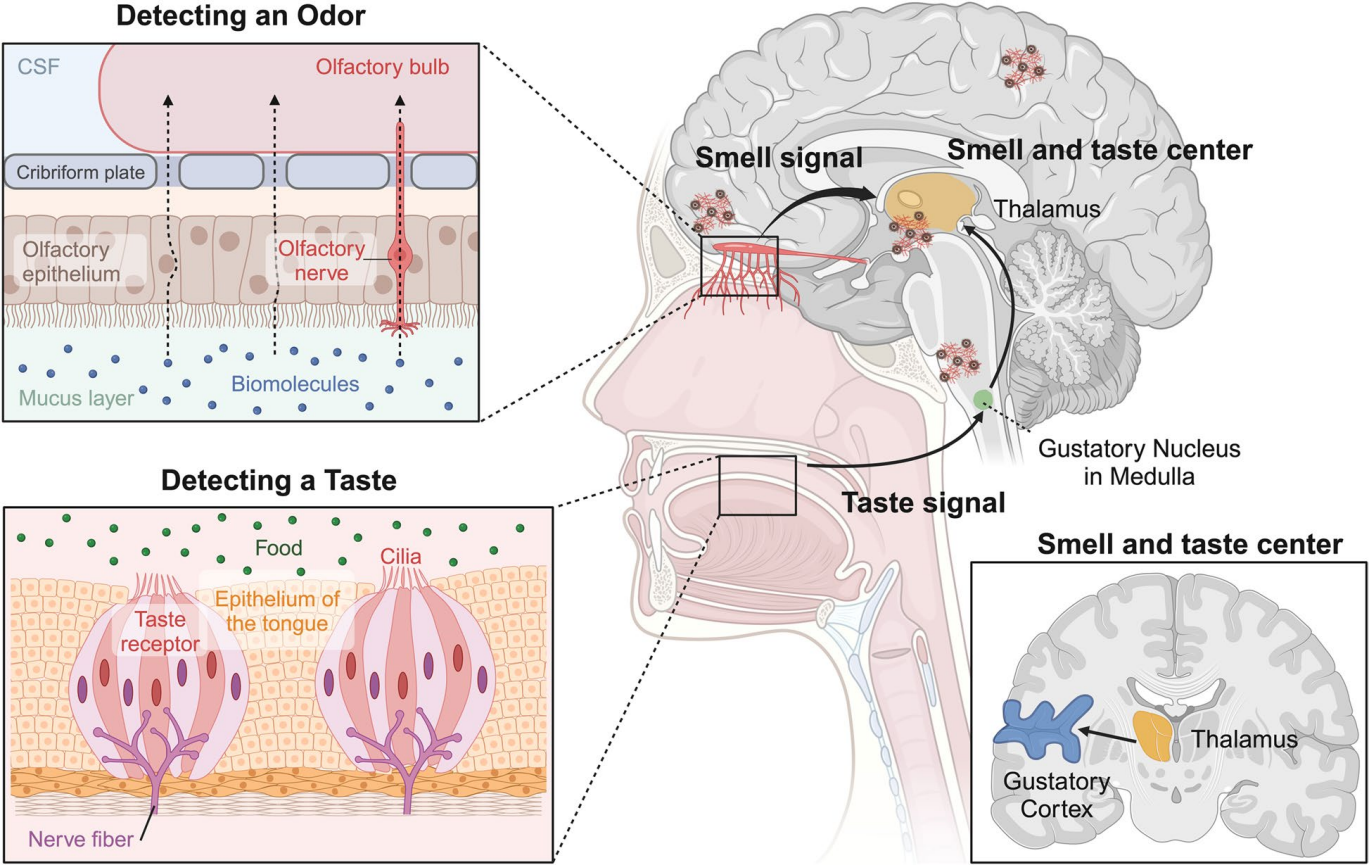
Descriptive Anatomy of Odorant Signal Transmission and the Interconnection with the Human Gustatory System in the Context of AD Pathogenesis. The intricate connections revealed in this illustration shed light on the olfactory and gustatory pathways, mapping their course from peripheral nerves to cortical areas. These connections further our comprehension of these sensory pathways within the intricate context of AD pathology. Image created using Biorender

Flavor perception arises from the central integration of various sensory inputs, including taste, smell, texture, temperature, visual, and auditory characteristics associated with food [219]. Psychophysical research and neuroimaging studies in humans, supplemented by electrophysiological data from animal models, are progressively revealing the neural foundations of flavor processing [220, 221]. Neuroimaging studies focused on taste and smell reveal that isolated exposure to tastants or odorants activates specific regions of the insula [222–225]. These insular regions are associated with primary taste perception, the amygdala involvement [222, 226, 227], which is pivotal for emotional processing, and the OFC [222–224, 228, 229], which plays a role in advanced taste and olfactory processing. Additionally, the anterior cingulate cortex is implicated in these complex sensory processes [230]. Figure 6 highlights the distinct yet overlapping neural pathways involved in taste and olfactory processing. Understanding these mechanisms is key to addressing the challenges posed by gustatory disorders, especially in the aging population.

### The gustatory system and AD

Recent studies have highlighted a notable pattern of diminished gustatory performance in patient, specifically in those with subjective cognitive decline [231]. This trend is also observed in individuals diagnosed with MCI and mild AD, suggesting a consistent pattern of gustatory dysfunction across these populations [232–234]. Research indicates that individuals with AD often show impaired gustatory function. This impairment is characterized by elevated thresholds for detecting and recognizing various tastes, including umami, sweet, salty, bitter, and sour. For instance, patients with AD commonly experience difficulties in recognizing umami flavors and detecting sweet and salty tastes [232, 235]. In contrast, some studies have found that older subjects with moderate dementia due to AD demonstrate significant reductions in taste recognition, particularly for bitter and salty tastes, compared to cognitively intact individuals [236]. These challenges suggest that there are deficits in taste processing within the neural system of patients with AD, evidenced by elevated thresholds for detecting and recognizing various tastes. This suggests that gustatory assessment could serve as a potential diagnostic tool for distinguishing patients with AD from their healthy individuals [237].

In addition, studies conducted by Kouzuki and colleagues (2018 and 2020) present divergent perspectives on the relationship between gustatory function and neurodegenerative conditions. The 2018 study observed no significant differences in gustatory test scores among AD, MCI, and healthy control groups. This implies that gustatory function may remain relatively intact in the AD and MCI [238]. In contrast, the 2020 study by the same group reported impaired gustatory function in patients with AD. For MCI patients, the results were inconclusive, but a noteworthy number had trouble in recognizing umami. This inconsistency underscores the intricate nature of gustatory changes in AD and emphasizes the imperative for further in-depth investigation [233].

### Potential mechanisms linking gustatory system and AD

The gustatory system is closely linked with brain areas affected early in AD, such as the hippocampus, amygdala, and orbitofrontal cortex, which are necessary for taste processing, memory, and emotional regulation [21]. The orbitofrontal cortex, vital for processing taste, shows early neurofibrillary tangle pathology in AD [239]. Damage here can impair taste perception and affect behaviors and decision-making, potentially accelerating cognitive decline. Furthermore, the insula, amygdala, and hippocampus, which are involved in taste processing, are also important in the context of AD [240, 241]. These regions are part of the limbic system, which integrates sensory information, including taste [242]. Disruptions in the gustatory pathways within these areas could influence the progression of AD by affecting memory and emotional processing. Volume changes in these regions, particularly in the MTL structures, might contribute to cognitive decline, suggesting that gustatory pathway dysfunctions could have a cascading effect on AD development. Neuroimaging studies, such as MRI, have revealed significant volume reduction in critical brain areas involved in memory and spatial navigation, including the hippocampus and entorhinal cortex, in individuals with MCI [243]. Furthermore, FDG-PET imaging has highlighted decreased glucose metabolism in the posterior cingulate cortex among MCI group, indicating early signs of neural dysfunction that precede AD [244, 245]. Recent studies using tau-PET imaging have further elucidated the early deposition of tau pathology in the MTL and posterior cingulate cortex of patients with AD [246]. This progression aligns with the known stages of tau pathology in AD, starting from the transentorhinal region and extending to other brain areas.

Changes in neurotransmitter systems, including acetylcholine and serotonin, are associated with both taste perception and cognitive functions [247, 248]. In AD, disruptions in these systems might originate from or be exacerbated by gustatory pathway dysfunctions [249]. The involvement of the cholinergic system in cognitive functions suggests that impairments in taste processing could have downstream effects on cognitive decline. Similarly, changes in serotonin receptors, which are involved in cognitive mechanisms, may be influenced by gustatory dysfunction, potentially accelerating AD progression [250].

The gustatory system, which processes taste sensations, shares similarities with the olfactory pathway in terms of neurological processing. Gustatory fibers might cross paths at the lower midbrain level, a feature reminiscent of the neural architecture of the olfactory system. Importantly, the pathways responsible for conveying taste-related information are closely linked with neural circuits extending to the amygdala and hippocampus (Fig. 6). These brain regions are not only pivotal in emotional responses and memory formation but are also implicated in the integration of taste sensations [251]. This neural overlap suggests that gustatory dysfunction could be related to the broader neural degeneration seen in AD, or potentially arise from various neurodegenerative processes contributing to MCI. In other words, the involvement of the amygdala and hippocampus in taste processing suggests that declines in gustatory function may reflect early neural changes occurring in these key brain regions.

#### Gustatory impairment and dementia: significance and future directions

The exploration of gustatory impairment as a biomarker for AD and related neurocognitive disorders (NCDs) is becoming increasingly important in the realm of diagnostic advancements. However, the relationship between gustatory thresholds and AD progression remains nuanced, requiring further elucidation through targeted research. A critical analysis integrating clinical diagnoses with biomarker evaluations of AD highlighted that no significant variance in gustatory thresholds exists between patients with amnestic MCI, those with AD dementia, and control groups [238]. This observation underscores the complexity of directly correlating taste performance with AD-specific biomarkers like CSF Aβ_42_ and phospho-tau levels. The lack of a direct association between these biomarkers and gustatory function underscores the complexities of sensory impairments in AD, calling the need for refined diagnostic criteria that consider the intricate nature of the disease. Given these insights, future directions in research should focus on delineating the specific aspects of gustatory dysfunction that correlate with AD progression. This entails not only the development of standardized gustatory testing protocols that can accurately measure taste impairments but also the examination of how these impairments interact with established clinical biomarkers of AD.

Further investigations employing chemical and electrical testing methods have uncovered gustatory dysfunctions specific to chemical stimuli in patients with AD, as opposed to electrogustometry, highlighting the variability in sensory testing outcomes [252]. Moreover, analyses of National Health and Nutrition Examination Survey data have established a significant link between the inability to identify salty tastes and dementia, further illustrating the specific taste impairments associated with cognitive decline [253]. Likewise, investigating the differential impact of AD on various taste qualities, such as sweet, salty, bitter, and umami, could provide deeper insights into the neurodegenerative processes underlying sensory alterations. Understanding the mechanisms through which AD affects the gustatory system, in conjunction with other sensory modalities, will be important in developing comprehensive diagnostic tools and therapeutic interventions aimed at improving the quality of life for individuals with AD.

Considering these insights, future research should aim to delve deeper into the specific aspects of gustatory dysfunction that align with progression of AD. This includes exploring the differential impact of AD on various taste qualities and how these changes interact with established clinical biomarkers. By adopting a more targeted approach to understanding gustatory impairments in AD, we can enhance diagnostic accuracy and potentially uncover novel pathways for early intervention and management of these complex neurodegenerative diseases.

## Tactile impairment

Tactile impairment is prevalent among older adults, with studies indicating that about 70% of individuals over the age of 70 experience some degree of touch impairment [254]. This impairment affects social interactions by reducing the ability to engage in social touch, such as handshakes or hugs, which are important for emotional connections [255]. As tactile sensitivity decreases with age, older adults often face challenges in performing tasks that require fine motor skills, such as buttoning clothes, tying shoelaces, or handling small objects [256]. This decline can lead to a loss of independence in carrying out basic activities of daily living and instrumental activities of daily living, including cooking, cleaning, and maintaining personal hygiene.

Decreased touch sensitivity results from changes in skin elasticity and nervous system function, making it more difficult for the brain to process touch signals [257]. This reduction can lead to decreased sensitivity to temperature, pressure, and pain, increasing the risk of injuries like burns or cuts, as individuals may not perceive these dangers promptly [258, 259]. Diminished pressure sensitivity also heightens the risk of falls and pressure ulcers, further compromising safety and affecting balance and bodily awareness. This can result in social withdrawal and isolation, impacting mental health and increasing the risk of depression. Furthermore, impaired touch can hinder communication, as individuals may struggle with using technology that requires touch inputs, making it difficult to stay connected with family and friends and contributing to social isolation [255].

### The tactile system

**Fig. 7:**
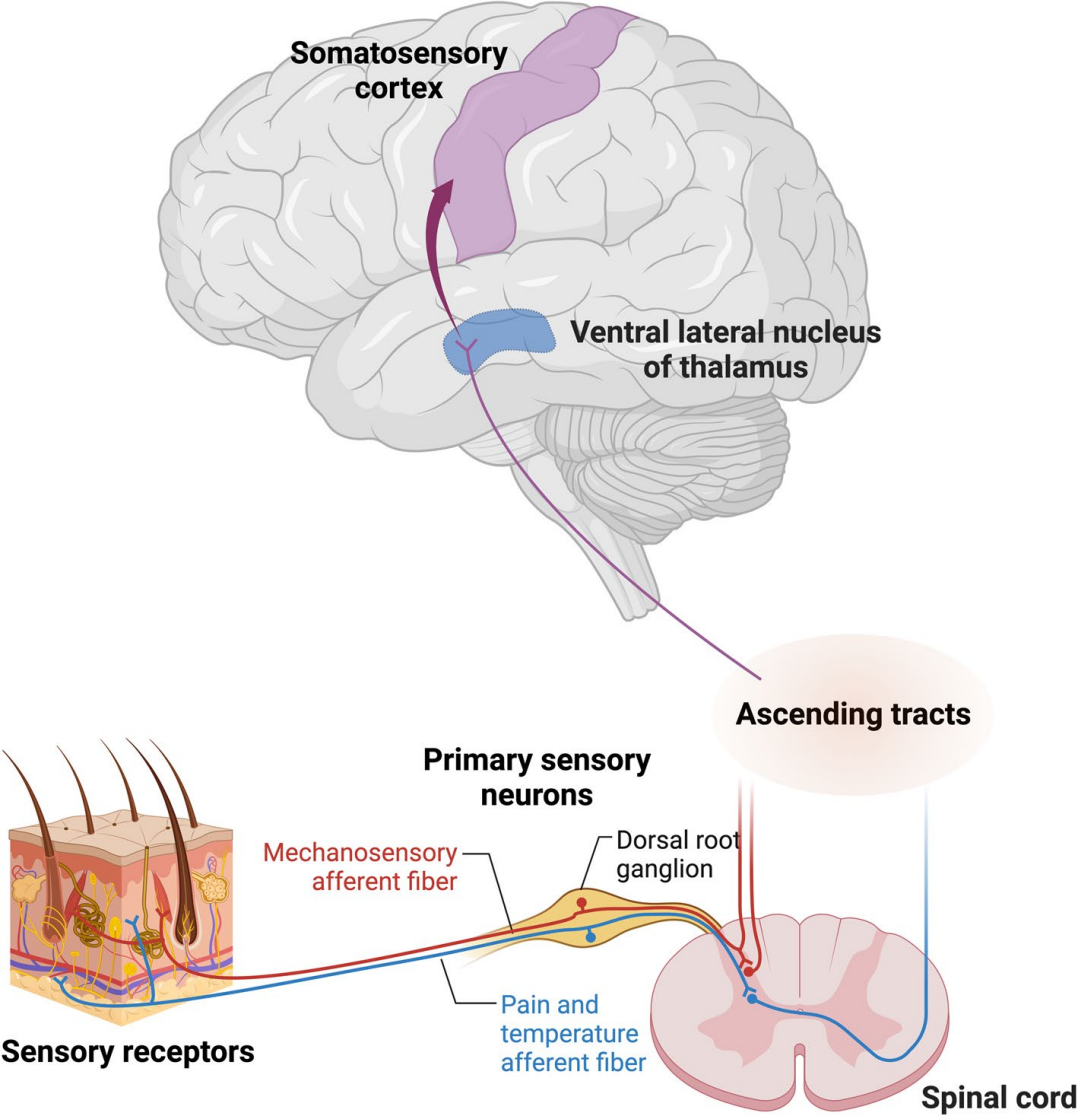
Schematic representation of major tactile pathway. Fibers conveying fine touch and proprioceptive sensations consolidate in the dorsal column of the spinal cord, ascending to the medulla oblongata. Those originating from the head unite with fibers from the rest of the body in the brainstem region, following the same path to the cerebral cortex. Image created using Biorender

The tactile system, distinct from other sensory systems, exhibits an extensive distribution throughout the body, relying on sensory receptors in the skin for information acquisition. This perceptual modality involves sensing physical contact and pressure on the skin, constituting an integral aspect of the broader somatosensory function [260]. The somatosensory system, responsible for the conscious perception of tactile, thermal, nociceptive, proprioceptive, kinesthetic, and vibratory sensations, receives inputs from various peripheral sources, including musculature, joints, cutaneous structures, and fascial tissues (Fig. 7). The somatosensory system operates through a three-neuron relay mechanism [261] Sensory signals originating from peripheral receptors travel through sensory afferents to the dorsal root ganglia, where the first-order neuron cell bodies reside. These first-order neurons then progress through the spinal cord, intersecting with second-order neurons, whose location varies depending on the specific sensory modality they mediate. The spinal cord houses second-order neurons for processing pain, touch, and temperature signals, while the medulla contains neurons specialized in transmitting tactile, proprioceptive, and vibratory information. Sensory fibers branch into pathways towards the thalamus or the cerebellum, both of which predominantly manage unconscious information processing. The thalamic projections are vital for conveying sensory data to cortical regions, where complex sensory integration and analysis contribute to conscious perception.

Despite its importance, the body of experimental and clinical evidence concerning age-related changes in the somatosensory system remains equivocal [34, 262]. This ambiguity in findings may be attributed to the intricate interaction of the central and peripheral nervous systems, along with the skin, in processing somatosensory information [263–265]. Understanding the nuanced interplay among these components is essential for elucidating the complexities of age-related alterations in tactile perception and the broader somatosensory system.

It is noteworthy that somatosensory acuity varies significantly among individuals and changes throughout the lifetime [266]. The aging process is inherently linked with a noticeable decline in motor and somatosensory functions [267, 268]. These age-related somatosensory changes in can impact tactile perception. As individuals age, they often experience alterations in cutaneous sensitivity, affecting their ability to perceive and distinguish tactile stimuli [269]. Primarily, aspects of age-related motor decline have been causally linked to reductions in inhibitory function, highlighting a mechanistic aspect. These changes include reductions in brain volume, alteration in patterns of brain functional activity and connectivity, and decreased neurotransmission levels [270–274]. Consequently, it is essential to acknowledge that age-related alterations in sensory perception and motor function might contribute substantially to the heightened vulnerability to AD and other age-related neurodegenerative diseases. Understanding these changes is important for developing comprehensive approaches to managing and treating AD, where sensory and motor functions are integral to the life satisfaction of patients and the effectiveness of therapeutic interventions.

### The tactile system and AD

The relationship between the tactile system and AD is an area of growing interest in neuroscience research. Research indicates significant alterations in somatosensory perception and processing among patients with AD, including diminished responsiveness to tactile stimuli. This reduced sensitivity affects their ability to perceive discomfort, pain, and temperature changes. Furthermore, these individuals often struggle to differentiate between various textures and other sensory inputs [275–277]. Studies comparing patients with AD with age-matched controls have found that sensory impairments are more severe in patients diagnosed with AD. For instance, an initial clinical study involving 21 patients with AD, and 15 age-matched controls revealed that patients with AD demonstrated distinct tactile deficits. They made significantly more errors during tactile pattern recognition tasks, with error rates approximately four times higher than those observed in non-affected individuals [278]. Another investigation, involving 40 participants (15 with AD, 10 with MCI, and 15 controls) highlighted similar findings in tactile angle discrimination, showing significant deficits among the AD and MCI groups compared to the controls [276]. Expanding on these observations, a larger study included 120 participants divided into four groups: normal controls, individuals with subjective cognitive decline, those with amnestic MCI, and patients with AD. The study demonstrated that accuracy in tactile angle discrimination progressively worsened from the control group through to the patients with AD, confirming a gradual decline in tactile abilities as part of the AD progression [279].

Tactile stimulation has been found to alleviate AD symptoms in mouse models. Specifically, these models exhibit improvements in cognition, such as enhanced performance in the Morris water maze test. Additionally, tactile stimulation has been shown to improve motor functions, as evidenced by better performance in the rotarod test, and to reduce anxiety-like behaviors, observed through decreased time spent in the open arms of the elevated plus maze. These behavioral improvements are accompanied by a reduction in AD pathology, including decreased Aβ plaque deposition and tau phosphorylation [280]. In humans, a 6-week tactile massage therapy regimen administered to elderly individuals with dementia resulted in decreased aggression and stress levels, suggesting improvements in physiological and psychological functioning [281]. Some studies have found impairments in tactile perception in individuals with AD, which may contribute to cognitive and functional decline. However, the exact nature of this relationship and its implications for disease management require further investigation. To establish an important role for tactile stimulation in AD management and disease progression, larger-scale clinical trials in humans with AD are necessary. These studies should examine the effects of various tactile interventions on cognitive function, behavior, quality of life, and biomarkers of disease progression over extended periods. Additionally, research into the underlying mechanisms by which tactile stimulation might influence AD pathology is needed to support any claims about its potential therapeutic value. This could include investigations into how tactile input affects neural plasticity, inflammation, or other relevant physiological processes in the context of AD.

### Potential mechanisms linking tactile function and AD

The mechanisms underlying the relationship between tactile sensory function and AD are still being investigated. Recent studies suggest that tactile stimulation may have positive effects on cerebral and behavioral development, offering insights into novel approaches for managing AD [282–285]. Tactile stimulation has been found to trigger several beneficial neurobiological processes. One key mechanism involves the release of neurotrophic factors. Specifically, tactile stimulation has been shown to induce the epidermal release of fibroblast growth factor-2 (FGF-2) [286], which initiates a cascade of biological processes including neurogenesis, cellular proliferation, cell survival, migration, and neural differentiation [287]. Additionally, increased levels of brain-derived neurotrophic factor (BDNF) have been observed following tactile stimulation, contributing to enhanced synaptic plasticity and neuronal survival [288]. Another important aspect of tactile stimulation is neurotransmitter modulation. Research has associated tactile stimulation with increased levels of acetylcholine [289], a neurotransmitter critical for cognitive function and memory formation. This modulation may play a role in improving cognitive outcomes in patients with AD. Synaptic plasticity, fundamental to learning, memory, and cognitive function, has also been shown to be enhanced by tactile stimulation [290, 291]. This enhancement could potentially contribute to cognitive resilience in individuals at risk for or diagnosed with AD.

Studies in rodent models have demonstrated that tactile stimulation aids in post-brain trauma recovery and reduces Aβ plaque deposition and tau phosphorylation, suggesting a potential neuroprotective effect in AD [280, 285]. This direct impact on AD pathology suggests that tactile stimulation could potentially slow disease progression. This neuroprotective capacity is further supported by observations in preterm infants, where tactile stimulation has been associated with enhanced cognitive and motor skills, indicating a potential for improving brain function through tactile interventions.

While not explicitly demonstrated in current research, it is hypothesized that tactile stimulation may also influence neuroinflammatory processes, which are known to play a role in AD progression [285]. Furthermore, tactile stimulation may enhance overall sensory integration and provide cognitive stimulation, potentially contributing to cognitive reserve and resilience against AD progression. These findings collectively suggest that tactile stimulation could be a promising non-invasive strategy for slowing the onset of dementia in aging individuals. However, it is necessary to note that while these results are encouraging, further research is needed to fully understand the mechanisms involved and to develop effective interventions for patients with AD.

#### Tactile impairment and dementia: significance and future directions

Current research is intensively exploring tactile impairments and their correlation with cognitive deficits in AD [45, 279, 280, 285, 292–294]. Recent findings suggest that tactile discrimination tasks, which assess the ability to distinguish stimuli through touch, may serve as effective indicators of cognitive decline in individuals with MCI due to AD. These tasks are advantageous because they are quick, easy to compare, and less influenced by educational background, offering a potential method for early diagnosis and intervention [279, 295]. While animal studies have provided insights into abnormal sensory processing in AD models, further human studies are necessary to fully understand how these sensory deficits affect brain function and disease progression in patients with AD [9, 292].

Efforts are also underway to create and validate standardized methods for assessing tactile function in individuals at risk of or exhibiting mild AD [296, 297]. This includes the development of tactile cognitive function tests that leverage both structural and metabolic information to improve early diagnosis [298]. Overall, these research initiatives highlight the importance of sensory processing in AD and underscore the potential of tactile assessments in clinical settings. Concurrently, efforts are underway to create and validate standardized methods and tools for accessing tactile function in individuals at risk of or exhibiting early-stage AD [296, 297].

The involvement of higher-order cortical sensory processing areas, particularly the parietal lobe, in AD has significant implications for sensory dysfunction [301]. The parietal lobe plays a pivotal role in integrating and processing somatosensory information, and its impairment in AD can lead to widespread effects on tactile recognition and spatial perception. Research has shown that patients with AD exhibit significantly higher error rates (approximately four times higher) in tactile pattern recognition tasks compared to healthy controls [278]. This suggests that parietal lobe dysfunction in AD affects not just simple sensory input processing but also complex tactile information interpretation and integration.

Efforts are also underway to create and validate standardized methods for assessing tactile function in individuals at risk of or exhibiting mild AD [296, 297]. This includes the development of tactile cognitive function tests that leverage both structural and metabolic information to improve early diagnosis [298]. Overall, these research initiatives highlight the importance of sensory processing in AD and underscore the potential of tactile assessments in clinical settings. Concurrently, efforts are underway to create and validate standardized methods and tools for accessing tactile function in individuals at risk of or exhibiting early-stage AD [296, 297].

Neurological pathways and brain regions affected by AD that contribute to tactile impairments are being investigated. Advanced neuroimaging techniques like MRI and PET scans have been employed to observe cerebral changes associated with tactile deficits [293, 299]. This includes examining how regions involved in sensory processing are affected in AD, particularly the impact of Aβ plaques and tau tangles on these areas. A deeper understanding of the cellular and molecular mechanisms underlying sensory deficits in AD could illuminate aspects of disease progression and reveal new therapeutic opportunities. Some studies are assessing the potential of tactile stimulation to mitigate cognitive decline in patients with AD. These involve sensory therapies, such as massages or the use of textured materials, designed to enhance cognitive and sensory engagement. The effectiveness of these interventions in improving quality of life, reducing agitation, and potentially slowing disease progression is a key area of research [280, 300].

The involvement of higher-order cortical sensory processing areas, particularly the parietal lobe, in AD has significant implications for sensory dysfunction [301]. The parietal lobe plays a pivotal role in integrating and processing somatosensory information, and its impairment in AD can lead to widespread effects on tactile recognition and spatial perception. Research has shown that patients with AD exhibit significantly higher error rates (approximately four times higher) in tactile pattern recognition tasks compared to healthy controls [278]. This suggests that parietal lobe dysfunction in AD affects not just simple sensory input processing but also complex tactile information interpretation and integration.

The relationship between sensory dysfunction in the elderly and metabolic polyneuropathy presents a complex challenge in establishing correlation and causation. While these two phenomena often co-occur, elucidating the nature of their relationship is not straightforward. The potential for underlying factors such as diabetes, hypertension, dyslipidemia, or nutritional deficiencies to affect both cognitive function and peripheral nerves further complicates this relationship [302]. It is important to consider that the primary contributors to dementia progression may not be sensory deficits themselves, but rather these underlying factors that impact both cognition and peripheral nerves. For instance, diabetes can lead to peripheral neuropathy, causing sensory dysfunction, while simultaneously affecting cognitive function through vascular damage in the brain [303–305]. This suggests that sensory dysfunction and cognitive impairment may be outcomes of shared pathological processes.

The complexity of unraveling the causal relationships between sensory dysfunction, cognitive impairment, and these potential shared risk factors or pathologies presents a considerable challenge. Longitudinal studies and multivariate analyses will be necessary to clarify these relationships. Additionally, neuroimaging techniques and biomarker analyses could provide more precise assessments of how these factors affect brain structure and function.

In conclusion, sensory dysfunction in AD is likely not merely a peripheral nerve issue but a result of higher-order cortical processing deficits, metabolic factors, and complex neurodegenerative processes. Understanding these intricate interactions could provide insights for early diagnosis and development of effective treatment strategies for AD. Future research should focus on disentangling these relationships to better understand the progression of AD and identify potential targets for intervention. As we explore the role of tactile sensation as a biomarker for AD, it becomes imperative to conduct in-depth studies into the relationship between tactile sensory changes and aging. Current hypotheses suggest that the measurement and manipulation of tactile sensation could play a pivotal role in the prevention, diagnosis, and treatment of AD, with future research expected to further solidify this potential.

### Multisensory deficits on alzheimer’s disease

Research has increasingly focused on how combinations of sensory impairments, particularly involving hearing, vision, and olfactory functions, impact AD. These studies collectively highlight the significant role that multisensory deficits play in increasing the risk of dementia and accelerating cognitive decline. Research has shown that individuals with dual sensory impairment (DSI) in hearing and vision have a significantly higher risk of developing AD compared to those with a single sensory impairment [306]. While some studies suggest that the risk is more pronounced with hearing loss [307], recent findings indicate a considerably higher hazard of dementia in individuals with combined sensory impairments dementia [308–311]. Further supporting this, *the Baltimore Longitudinal Study of Aging* explored the impact of multiple sensory impairments, including vision, hearing, olfactory, proprioceptive, and vestibular functions, on brain structure [312]. The study revealed that each additional sensory impairment was associated with lower volumes in critical brain regions like the orbitofrontal gyrus and entorhinal cortex. This indicates that the cumulative effect of multiple sensory deficits can lead to significant brain atrophy and cognitive decline. Similarly, the *Health*,* Aging*,* and Body Composition Study* created a summary measure of multisensory impairment based on vision, hearing, smell, and touch [313]. The findings demonstrated that worsening multisensory function was linked to a higher risk of dementia and faster rates of cognitive decline, emphasizing that even mild levels of multisensory impairment can accelerate cognitive aging. Olfactory impairment has been shown to significantly increase the risk of AD when combined with other sensory deficits such as hearing and vision loss [7, 200]. Olfactory dysfunction is linked to early neuropathological changes in brain areas like the olfactory bulb and entorhinal cortex, which are important for memory processing. When visual impairments are also present, they may compound the cognitive challenges, as both senses are essential for environmental interaction and memory cues. Moreover, another study found that individuals with multiple sensory impairments, including olfactory, hearing, and vision deficits, had a greater association with cognitive decline and dementia than those with a single sensory deficit [314, 315]. This suggests an additive effect where the combination of sensory impairments exacerbates the risk of AD.

In summary, multisensory deficits significantly impact AD by contributing to both the risk and progression of cognitive decline. Each sensory modality plays a role, and their combined effects highlight the importance of comprehensive sensory assessments and interventions in managing AD. Further research is needed to explore the mechanisms underlying these associations and to develop effective interventions targeting sensory impairments in patients with AD.

## Conclusions

Approximately 40% of individuals aged 70 to 79 experience dysfunction in at least one sensory system, with over 25% experiencing impairments in multiple senses [316]. These sensory changes often coincide with various comorbidities, specific to the affected sense. Notably, the presence of such sensory deficits is linked with an increased risk of developing AD. The exploration of age-related sensory organ impairments and their impact on AD carries substantial clinical relevance. Early identification and management of sensory impairments in the aging population could serve as a strategic approach to predict or mitigate cognitive decline associated with AD. Intervention targeting sensory deficits may not only improve the quality of life for these individuals but also potentially slow the progression of AD.

This review elucidates the complex interplay between sensory impairments and AD, highlighting the multifaceted nature of sensory decline as both an early indicator and a risk factor for AD. Auditory impairment, particularly age-related hearing loss, is strongly linked to increased cognitive decline and dementia risk, with hearing aids potentially mitigating cognitive deterioration. Olfactory dysfunction, correlating closely with cognitive decline and disease progression, emerges as a promising early biomarker for AD. Similarly, visual impairments, including retinal changes, are associated with an elevated risk of AD, positioning them as potential early indicators of neurodegeneration. Meanwhile, gustatory impairments, though less studied, show promise as markers for AD progression, particularly when occurring alongside other sensory deficits. Tactile impairments suggest broader impacts on somatosensory processing, affecting cognitive function and quality of life. This review further notes that multisensory deficits produce a cumulative effect, significantly increasing the risk of AD and accelerating cognitive decline beyond what is observed with single sensory impairments.

Moving forward, future research should prioritize several key areas to enhance our understanding and response to AD. It is essential to clarify the mechanisms that link sensory impairments to AD pathology, which could reveal new therapeutic targets. Developing standardized protocols for multisensory assessment will ensure consistent and early identification of at-risk individuals. Investigating sensory-based interventions offers potential new strategies for managing AD, possibly slowing or altering its course. This involves investigating the evolution of sensory deficits in the prodromal phase of AD, analyzing their timing in relation to CSF biomarkers, and utilizing measures from structural and functional MRI. Finally, conducting longitudinal studies is crucial for understanding how sensory decline correlates with the onset and progression of AD over time. Such research endeavors are key to improving early detection and intervention strategies for AD, ultimately contributing to more effective management and potentially altering the course of AD.

## Data Availability

Not applicable.
